# GreenViT: A Vision Transformer with Single-Path Progressive Upsampling for Urban Green-Space Segmentation and Auditable Area Estimation

**DOI:** 10.3390/jimaging12020072

**Published:** 2026-02-10

**Authors:** Ziqiang Xu, Young Choi, Changyong Yi, Chanjeong Park, Jinyoung Park, Hyungkeun Park, Sujeen Song

**Affiliations:** 1 Department of Robot and Smart System Engineering, Kyungpook National University, 80, Daehak-ro, Buk-gu, Daegu 41566, Republic of Korea; xzq@knu.ac.kr; 2 Earth Turbine, 36, Dongdeok-ro 40-gil, Jung-gu, Daegu 41905, Republic of Korea; youngch5@naver.com (Y.C.); songsujeen69@gmail.com (S.S.); 3 Intelligent Construction Automation Center, Kyungpook National University, 80 Daehak-ro, Buk-gu, Daegu 41566, Republic of Korea; tpms1018@knu.ac.kr (C.P.); jinypark@knu.ac.kr (J.P.); parkhk@chungbuk.ac.kr (H.P.)

**Keywords:** image processing, remote sensing image, urban green space monitoring, deep learning, vision transformer

## Abstract

Urban green-space monitoring in dense cityscapes remains limited by accuracy–efficiency trade-offs and the absence of integrated, auditable area estimation. We introduce GreenViT, a Vision Transformer (ViT) based framework for precise segmentation and transparent quantification of urban green space. GreenViT couples a ViT-L/14 backbone with a lightweight single-path, progressive upsampling decoder (Green Head), preserving global context while recovering thin structures. Experiments were conducted on a manually annotated dataset of 20 high-resolution satellite images collected from Satellites.Pro, covering five land-cover classes (background, green space, building, road, and water). Using a 224 × 224 sliding window sampling scheme, the 20 images yield 62,650 training/validation patches. Under five-fold evaluation, it attains 0.9200 ± 0.0243 mIoU, 0.9580 ± 0.0135 Dice, and 0.9570 PA, and the calibrated estimator achieves 1.10% relative area error. Overall, GreenViT strikes a strong balance between accuracy and efficiency, making it particularly well-suited for thin or boundary-rich classes. It can be used to support planning evaluations, green-space statistics, urban renewal assessments, and ecological red-line verification, while providing reliable green-area metrics to support urban heat mitigation and pollution control efforts. This makes it highly suitable for decision-oriented long-term monitoring and management assessments.

## 1. Introduction

In recent decades, unprecedented human-driven urban expansion has caused considerable environmental challenges, including global climate perturbations, biodiversity loss, ecosystem degradation, and urban heat islands, which directly affect health and quality of life in city regions [1,2]. The urban heat island effect impacts human health, particularly by increasing the incidence and mortality rates of cardiovascular diseases, respiratory diseases, heat-related illnesses, and mental health disorders [3,4,5,6]. Importantly, urban heat islands and air pollution are often intertwined rather than independent stressors. Elevated temperatures can accelerate photochemical pollutant formation (e.g., ozone) and, together with stagnant urban meteorology, reduce pollutant dispersion, leading to co-exposure and compounded cardiopulmonary risks [5]. According to the WHO (World Health Organization) global air quality guidelines, air pollution levels in megacities such as Delhi and Beijing frequently exceed the WHO’s recommended limits. Another study has shown that air pollution results in 7 million premature deaths worldwide annually [7]. Urban green spaces are considered the most effective nature-based solutions to deal with these issues, as they not only serve as natural spaces but also provide critical ecosystem services such as air purification, temperature regulation, and biodiversity conservation [8]. Beyond aggregate ecological benefits, the distribution of greenery also matters: visible green exposure during daily commutes can differ across marginalized and formal settlements, highlighting the need for accurate and equity-aware green-space mapping to support inclusive urban planning [9]. The IPCC (Intergovernmental Panel on Climate Change)’s AR6 (Sixth Assessment Report) stresses the importance of these urban green infrastructures as not only a means of adaptation but also to improve urban resilience [10]. Recent advances in artificial intelligence are also reshaping urban planning practice through GeoAI-enabled monitoring, predictive analytics, and decision support. Recent systematic reviews highlight the growing role of AI in urban design and planning workflows, including automated extraction of geospatial indicators and data-driven assessment of urban sustainability. In parallel, the GeoAI literature is rapidly expanding toward foundation-scale models and more explainable spatial learning, while planning-oriented studies increasingly connect machine learning outputs to actionable evaluation and policy decisions. These trends make accurate, scalable, and auditable green-space mapping an important building block for AI-assisted planning, because green infrastructure indicators are frequently used to assess climate adaptation, environmental exposure, and long-term urban management [11,12,13].

Traditional urban green-space monitoring faces accuracy–efficiency trade-offs: manual surveys are informative but time-consuming and error-prone [14], while early pipelines based on color-space heuristics (e.g., HSV thresholding; NDVI/ExG) and classical machine learning (hand-crafted texture + SVM/RF) are brittle under illumination changes and scale variability in dense urban scenes [15,16]. With high-resolution satellite imagery, CNN-based semantic segmentation (e.g., U-Net/DeepLab/HRNet) has achieved notable success [17,18,19], yet performance often degrades on thin or small green strips because of limited long-range context and an emphasis on local features [20]. Vision Transformers (ViTs) leverage self-attention to capture global dependencies [21]. Recent remote-sensing segmentation frameworks also explore hybrid CNN–ViT designs to combine CNN inductive biases for local detail with transformer-based long-range interactions; for example, CTSeg collaboratively learns CNN and ViT representations for high-resolution land-use/land-cover mapping [22], and comparative studies show advantages over CNNs in modeling long-range spatial relations, global context understanding, scalability, and adaptability [23]; recent variants such as Swin-T and SegFormer-Tiny provide competitive alternatives for complex urban layouts. Nevertheless, the joint problem of high-precision segmentation with reliable area quantification remains understudied in urban green-space monitoring [24]. Against this backdrop, we propose GreenViT, which couples a ViT backbone with a single-path, progressive multi-stage upsampling head and a fixed-weight composite loss, explicitly targeting class imbalance and boundary fidelity, and further provides a pixel-to-physics calibration pipeline that converts pixel counts into physical area estimates.

This study investigates an enhanced ViT-based framework that employs a single-path, progressive multi-stage upsampling decoder to achieve high-precision segmentation and quantification of urban green spaces. Specifically, the primary contributions of this study include:

- GreenViT Head for ViT token grids. We propose GreenViT on a ViT-L/14 backbone. Starting from the 16 × 16 token lattice (ViT-L/14), the single-path decoder performs progressive upsampling (×2, ×2, ×2, ×1.75) to align with the 224 × 224 image resolution, using lightweight convolutional blocks (1 × 1 → 3 × 3 + BN + ReLU) at each stage to refine boundaries. This design avoids multi-branch/multi-scale feature fusion, preserves the ViT’s global receptive field, and reconstructs a token-to-pixel semantic map with stable thin-structure recovery.
- Fixed-weight composite loss for class imbalance and boundary robustness. We adopt a fixed-weight loss 0.4 × CE + 0.3 × Dice + 0.2 × Focal(γ = 2) + 0.1 × Tversky(α = 0.7, β = 0.3) to handle sparse foregrounds and skewed scale distributions, reducing false positives/negatives while improving contour fidelity.
- Pixel-to-physics calibration and consistency evaluation. We provide an end-to-end procedure to convert segmentation pixels into physically meaningful area estimates using per-image scale calibration. The pipeline reports absolute and relative area errors between predictions and ground truth without relying on external GIS software.

Different from common remote-sensing segmentation pipelines that rely on multi-scale feature pyramids and skip-connected decoders (e.g., U-Net/FPN-style designs), GreenViT is built around a pure-ViT token lattice and a single-path progressive upsampling head that directly maps tokens to pixels through lightweight refinement blocks. This design deliberately minimizes decoder branching and feature fusion, keeping the data flow simple and reproducible while targeting boundary-rich urban scenes. In addition, we integrate an explicit pixel-to-physics calibration stage (mask → pixel counting → calibrated conversion) so that the framework outputs not only semantic metrics but also auditable area estimates. Together, the decoder design and calibration pipeline form a compact end-to-end methodology tailored to high-precision green-space monitoring with measurable, decision-oriented outputs.

## 2. Related Works

Urban green-space monitoring has been approached through a wide spectrum of methods that differ in sensing assumptions, model capacity, and deployment cost. To situate our approach and enable fair comparison, we review this landscape along a progression from rule-based pipelines to modern Transformer models, highlighting how each family addresses challenges typical of dense urban scenes.

### 2.1. Traditional Methods for Urban Green Monitoring

Early urban green monitoring relied on manual surveys and simple color-space heuristics such as HSV thresholding and vegetation indices. In dense urban scenes, however, the complex three-dimensional structure of buildings and trees yields extensive cast shadows and within-scene illumination variability, which can severely bias RGB-based indices and fixed thresholds. A recent review of urban vegetation mapping highlights that urban spatial–spectral heterogeneity and 3D structure lead to large, shadowed areas and radiometric effects, making optical delineation of vegetation challenging [25]. Consistent with this, Lin et al. (2023) reported that building shadows remain a major source of omissions and misclassification in high-resolution urban vegetation extraction [26]. L. Yang et al. (2022) found that when applying HSV color space and ExG (Excess Green) thresholding methods to digital images, the use of fixed thresholds often leads to severe misclassification under conditions of strong shadows or water stress [27]. Z. Luo et al. (2024) further noted that along shadow boundaries or under strong illumination gradients, shadows can significantly disrupt the reliable interpretation of remote sensing imagery and degrade recognition performance [28].

### 2.2. Classical Machine Learning Approaches

Classical machine learning approaches combine hand-crafted descriptors (color/texture/shape) with SVM (Support Vector Machine) or Random Forest classifiers. While they reduce part of the brittleness of pure thresholding, their performance typically depends on careful feature design and may generalize poorly under the strong heterogeneity, scale variance, and shadowing present in high-resolution urban mosaics [25]. For urban vegetation extraction, Lin et al. (2023) compared deep learning segmentation with NDVI- and Random Forest-based baselines and reported clear accuracy advantages of deep models, indicating that shallow hand-crafted pipelines often struggle in complex urban scenes [26]. These limitations motivate end-to-end representation learning for robust urban green-space segmentation.

### 2.3. Deep Learning Methods for Semantic Segmentation

Due to the success of Deep Convolutional Neural Networks (DCNNs) in academic and industrial domains, semantic segmentation methods based on DCNNs are receiving increasing attention in the interpretation and information extraction of remote sensing imagery [29]. For urban vegetation and green-space delineation, encoder–decoder architectures (e.g., U-Net and DeepLab variants) can effectively exploit local texture and edge cues, but limited receptive fields and pooling/stride-induced smoothing often blur thin strips (e.g., narrow medians and street-tree belts) and soften boundaries, especially under strong class imbalance and shadow-induced radiometric variation. Prior work has explored multi-scale context modules (e.g., atrous spatial pyramid pooling), feature fusion, and loss re-weighting to recover fine structures, yet long-range contextual reasoning and global shape consistency remain challenging for purely convolutional designs. Recently, hybrid networks have begun to integrate multi-scale feature fusion with transformer-style global modeling for urban green-space segmentation; for example, Cheng et al. proposed MFFTNet, which couples a transformer network with multi-scale fusion (and optional vegetation cues) to improve edge recovery in high-resolution urban green-space datasets [30]. These advances motivate our shift toward transformer-based global context modeling while using a lightweight decoder to restore boundary details.

### 2.4. Transformer-Based Segmentation Methods

Recent studies show that transformer-based models have significant potential in capturing global dependencies. By leveraging multi-head self-attention mechanisms, transformer-based models can handle long-range contextual information, demonstrating strong performance in dealing with complex remote sensing images [31,32]. Wang et al. (2022) showed that a transformer-based model can help improve segmentation accuracy in complex backgrounds [33], Aleissaee et al. (2023) showed transformers in remote sensing capture long-range dependencies through self-attention, overcome CNN limitations, offer strong feature representation, and, when combined with CNN in a hybrid architecture, provide flexible adaptivity and good performance even with limited data [34]. However, many transformer-based decoders tend to focus on large-scale structures, leading to insufficient representation of minority classes and fine-grained objects [35]. In addition, the high computational cost of self-attention makes it difficult to process high-resolution images in real time on edge devices, thereby limiting their practical applicability. Therefore, the application of traditional transformer-based methods to urban green space segmentation and precise area quantification remains underexplored. Existing transformer-based models often emphasize segmentation metrics alone, with fewer studies incorporating geospatial calculations needed for urban ecology and planning. Additionally, concerns regarding computational cost and efficient training strategies persist, particularly when handling ultra-high-resolution satellite imagery.

### 2.5. Research Gaps, Challenges, and Motivation

Recent deep learning advancements have effectively enhanced segmentation performance for remote sensing images, yet notable challenges remain, particularly regarding comprehensive capture of global context, quantifiable outcome integration, and reliable deployment in heterogeneous urban environments. Traditional convolution-based methods often fall short at capturing extended spatial relationships, hindering the accurate delineation of fragmented or irregular vegetation patches in cityscapes. Furthermore, most existing frameworks lack an embedded process for quantitative area measurement, limiting their practical applicability to urban planning scenarios. Beyond these CNN-centered limitations, urban areas themselves pose unique complexity: buildings, roads, and water bodies intermingle with sparse, differently lit, or partially obscured green spaces, skewing class balance and complicating consistent model reliability. Transformer-based methods such as ViTs, with their capability to model long-range dependencies via multi-head self-attention, promise a more holistic feature representation capable of handling this diversity. Yet, despite the potential to incorporate global context, research on leveraging transformers explicitly for green space detection with integrated measurement is still underexplored. These gaps motivate our ViT-backbone design with a lightweight single-path, progressive upsampling decoder and targeted loss design. By harnessing transformer-driven global modeling and purposefully coupling it with quantification, this approach aspires to offer a robust solution to the pressing challenges in precise urban green space segmentation and measurement.

## 3. Methods

This section describes our ViT-based segmentation system and the design choices that make it both accurate and practical. The core idea is to keep the backbone untouched and decode the patch-token grid with a single-path, progressive upsampling head. We also employ a fixed-weight composite loss that explicitly targets class imbalance and boundary fidelity.

### 3.1. GreenViT Architecture

**Figure 1:**
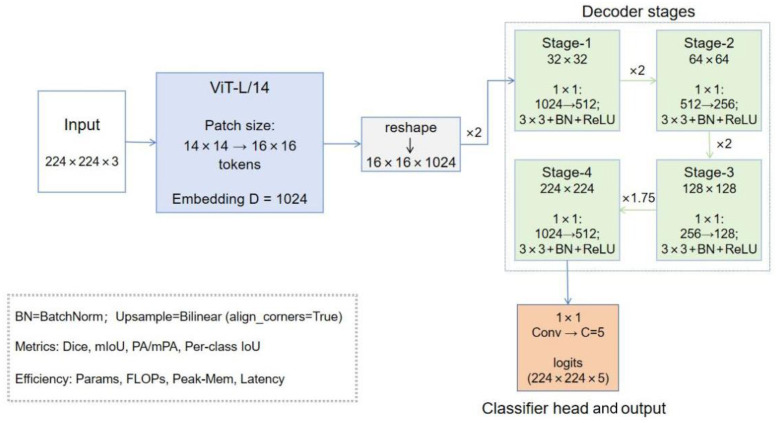
Overview of GreenViT.

Our GreenViT preserves the strong global reasoning capability of the ViT encoder while adopting our self-developed lightweight progressive upsampling head (GreenViT Head) to replace bulky decoders. Starting from ViT-L/14 patch tokens (16 × 16 tokens at 224 × 224 input, each 1024-D), we reshape tokens back to a grid and decode to full resolution through four compact stages. Each stage upsamples the feature map and refines it with a 1 × 1 bottleneck (for channel reduction) followed by a 3 × 3 local refinement (for boundary detail), using BatchNorm and ReLU. The design is intentionally simple: no feature pyramid from the encoder, no learned upsampling, minimal memory footprint, and exact output size recovery (16 → 32 → 64 → 128 → 224). Compared to a naive “linear projection + single upsample” ViT segmentation head, GreenViT integrates multi-scale local features while preserving the encoder’s global context, enhances boundary precision through shallow spatial convolutions, and maintains efficiency with a low parameter count and fast inference. Its overall architecture is shown in Figure 1. In CNN encoders, skip connections are natural because early layers provide high-resolution feature maps. In a pure ViT, features are represented primarily on a single token grid; constructing a multi-level pyramid typically requires additional re-embedding, pooling, or extra feature taps, which increases architectural complexity and memory usage. Our decoder instead progressively refines the token grid to recover spatial detail, leveraging the ViT’s global context while adding only lightweight local convolutions to sharpen boundaries. This choice reduces branching and fusion nodes, lowers engineering overhead, and keeps the method easier to reproduce and diagnose (errors remain attributable to the backbone and decoder rather than post hoc fusion heuristics).

### 3.2. Token-to-Grid Projection

Let the ViT backbone output token embeddings $X\in R^{B\times S\times D}$, with $S=P^{2}$ and D=1024 for ViT-L/14 at 224 × 224. We retain the backbone’s native positional encoding and discard any class token, only the S patch tokens are used for decoding. To restore spatial layout, we reshape the sequence into a feature grid as in Equation (1)

(1) $$F_{0}=reshape(X)\in R^{B\times D\times P\times P},P=\sqrt{S}=16$$

This step restores spatial topology while preserving the encoder’s global context. It also decouples the decoder from the encoder internals; any ViT that returns patch tokens can be plugged in. We do not extract multi-scale features from intermediate ViT blocks. Instead, we decode from the single token grid, preserving the backbone’s global modeling while keeping memory and parameter overhead low.

### 3.3. GreenViT Head (Single-Path, Progressive Upsampling)

Unlike pyramid fusion, GreenViT adopts a single-path progressive refinement starting from the token grid, without any multi-branch fusion or skip concatenations. Given, the head restores resolution purely by bilinear upsampling (×2, ×2, ×2, ×1.75; align_corners = true) and lightweight conv refinements that repeat the same three-step pattern at each stage: (i) a 1 × 1 bottleneck to reduce channels, (ii) a 3 × 3 conv + BN + ReLU for local detail, and (iii) output resampling to the next spatial scale. Concretely, the schedule is as follows:

Stage 1 (×2): 1 × 1 Conv 1024 → 512 + ReLU; 3 × 3 Conv 512 → 512 + BN + ReLU → B × 512 × 32 × 32.

Stage 2 (×2): 1 × 1 Conv 512 → 256 + ReLU; 3 × 3 Conv 256 → 256 + BN + ReLU → B × 256 × 64 × 64.

Stage 3 (×2): 1 × 1 Conv 256 → 128 + ReLU; 3 × 3 Conv 128 → 128 + BN + ReLU → B × 128 × 128 × 128.

Stage 4 (×1.75): 1 × 1 Conv 128 → 64 + ReLU; 3 × 3 Conv 64 → 64 + BN + ReLU; final 1 × 1 Conv 64 → 5 to produce class logits, followed by bilinear resize to 224 × 224.

We intentionally avoid transposed convolutions, attention add-ons, residual/skip connections, or pyramid branches; the decoder is strictly bilinear upsampling + (1 × 1 reduce + 3 × 3 refine + BN + ReLU) blocks, which keeps the head lightweight while recovering thin structures.

### 3.4. Loss Functions (Fixed-Weight Composite Objective)

In order to handle the key problem of class imbalance in satellite imagery segmentation and enhance model performance, we propose a composite loss function that combines Cross-Entropy (CE), Dice Loss, Focal Loss, and Tversky Loss. To address class imbalance and sharpen boundaries while keeping comparisons fair across all backbones, we train with the same fixed linear combination for every model (GreenViT, Comparative experiment models: Swin-T, SegFormer-B0).

#### 3.4.1. Cross-Entropy (CE) Loss

Cross-Entropy (CE) provides standard pixel-wise multi-class supervision with well-calibrated probabilities and stable gradient signals, which can significantly improve convergence stability, especially in the early stages of training. CE enforces uniform, per-pixel constraints across all classes, preventing the gradient instability and early oscillations that may occur when optimizing solely for region-overlap-based objectives. Let $p_{t}$ denote the predicted probability of the true class (the entry of p indexed by y). Then CE Loss is defined as in Equation (2):

(2) $$L_{CE}=-\frac{1}{BHW}\sum_{b,h,w}\log p_{t}(b,h,w)$$

#### 3.4.2. Dice Loss

Dice shares the same formulation as common segmentation metrics (Dice/mIoU), directly optimizing region-level overlap quality. Compared with CE, Dice is more sensitive to small foreground targets and can effectively mitigate the learning bias introduced by foreground–background imbalance. The Dice Loss is defined as in Equation (3):

(3) $$L_{Dice}=1-\frac{1}{C}\sum_{c=1}^{C}\frac{2\sum p_{c}t_{c}+\epsilon }{\sum p_{c}+\sum t_{c}+\epsilon }$$

where $P_{c}$ and $t_{c}$, respectively, denote the predicted probability and the one-hot ground truth of the class c.

#### 3.4.3. Focal Loss

Focal Loss suppresses easy samples (high-confidence correct pixels) and amplifies the contribution of hard samples (low-confidence or misclassified pixels) by modulating their gradient magnitudes. This alleviates the issue of overwhelming background pixels dominating the loss and shifts the model’s focus toward boundaries, minority classes, and ambiguous regions. In our work, we set γ = 2. The Focal Loss is defined as in Equation (4):

(4) $$L_{Focal}=-\frac{1}{BHW}\sum_{b,h,w}{\left(1-p_{t}(b,h,w)\right)}^{\gamma }\log p_{t}(b,h,w)$$

#### 3.4.4. Tversky Loss

Tversky Loss is a generalized form of Dice that allows differential weighting of false positives (FP) and false negatives (FN). By adjusting these penalties, it provides a flexible trade-off that can be tailored to prioritize fewer false alarms or fewer misses, depending on the application. In this work, we set α = 0.7, β = 0.3, which biases the loss toward suppressing over-segmentation (reducing FP). The Tversky Loss is defined as in Equations (5)–(7):

(5) $${TP}_{c}=\sum p_{c}t_{c},\;{FP}_{c}=\sum p_{c}(1-t_{c}),\;{FN}_{c}=\sum (1-p_{c})t_{c}$$

(6) $${Tversky}_{c}=\frac{{TP}_{c}+\epsilon }{TP_{c}+\alpha {FP}_{c}+\beta {FN}_{c}+\epsilon }$$

(7) $$L_{Tversky}=1-\frac{1}{C}\sum_{c=1}^{C}{Tversky}_{c}$$

#### 3.4.5. Rationale and Complementarity of the Composite Objective

A single loss is often insufficient to simultaneously ensure stability, robustness to class imbalance, and sensitivity to both hard samples and task-specific trade-offs (FP/FN preferences). Therefore, we combine multiple complementary objectives:

- CE provides pixel-level supervision with consistent gradient signals, ensuring well-calibrated overall performance;
- Dice directly optimizes region-level overlap, performs well on small or minority classes, and mitigates imbalance;
- Focal down-weights easy samples and emphasizes hard ones, encouraging the model to focus on boundaries and ambiguous regions;
- Tversky, parameterized by (α,β), allows flexible trade-offs between precision and recall by reweighting FP and FN contributions.

From a gradient perspective, CE mainly enforces pixel-wise $p_{t}-1$ supervision, offering stable gradients across the image space; Dice and Tversky rely on region-level TP/FP/FN statistics, providing overlap-oriented optimization; focal adaptively amplifies the gradients of hard samples. Their complementary nature helps reduce the bias and instability associated with optimizing any single objective in isolation. Through experiments, we observed that when the weight assigned to CE falls outside the range of 0.35~0.45, early-stage oscillations become more pronounced under Dice/Tversky-dominated settings. When the Dice weight is set outside 0.25~0.35, training sometimes exhibits instability in the early phase or shows oversensitivity to low-probability noise. Similarly, assigning a Focal weight outside 0.15~0.25, particularly values above 0.25, causes training to be dominated by a very small fraction of hard pixels, slowing global convergence. Since Tversky primarily serves as a fine-tuning term for task-specific adjustments, our experiments demonstrate that setting α = 0.7 achieves a mild suppression of FP and improves precision, provided it does not strongly conflict with Dice or Focal. Accordingly, we adopt the composite objective like Equation (8):

(8) $$L=0.4L_{CE}+0.3L_{Dice}+0.2L_{Focal}\;(\gamma =2)+0.1L_{Tversky}(\alpha =0.7,\beta =0.3)$$

### 3.5. Data Preprocessing and Augmentation

Our data pipeline is designed to (i) expose the model to diverse local contexts and object boundaries without changing the network’s input geometry, (ii) preserve pixel-wise alignment between images and labels under augmentations, and (iii) match the ViT-L/14 backbone’s input statistics so that pretraining benefits transfer cleanly. These choices directly serve our accuracy metrics and the green area quantification.

#### 3.5.1. Sliding Window Sampling

**Figure 2:**
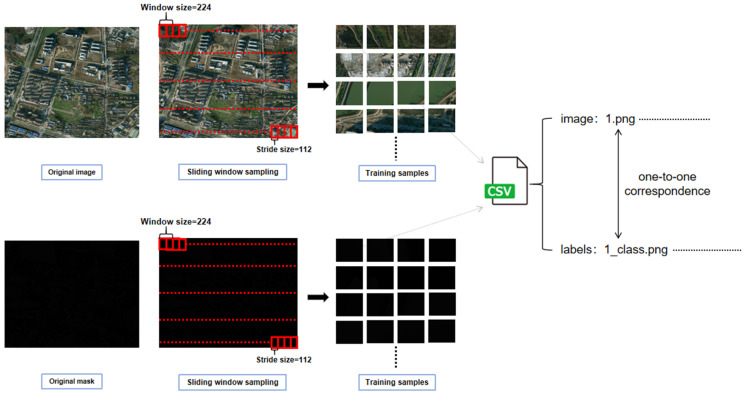
Illustration of the sliding-window sampling process used to generate training patches and maintain one-to-one image–label correspondences.

To systematically and uniformly obtain training samples from large-scale remote sensing imagery, we adopt a sliding-window cropping strategy with a default window size of 224 and a stride of 112. For each original image and its corresponding label map (read as a single-channel grayscale mask), we traverse the 2D grid to extract patches, saving the resulting sub-images into designated directories while maintaining a CSV (Comma-Separated Values) file that records the one-to-one correspondence between images and labels for subsequent dataset loading; the process is shown in Figure 2.

In our experiments, the implementation explicitly sets size = 224 and stride = 112 and employs a nested loop to perform patch extraction and batch writing to disk. Upon completion, a mapping list is written out via pandas. DataFrame, and the target directories are automatically created prior to execution to ensure reproducibility. This strategy is designed to substantially expand the sample pool, alleviate boundary fragmentation effects, and guarantee systematic coverage, all under a uniform input resolution. Compared with random cropping, sliding-window sampling covers the entire image without introducing sampling bias, offering several advantages: (a) unified input size ensures that subsequent networks (with 224 input) require no additional rescaling; (b) overlapping patches provide contextual redundancy during training, which stabilizes boundary predictions; (c) the CSV file that preserves image–label correspondences ensures transparent data lineage and enables reproducible experiments.

#### 3.5.2. Single-Channel Label Encoding and Consistency Handling

To avoid channel mixing introduced by color-encoded annotations during geometric transformations and rescaling, we read and write labels in a single-channel grayscale format at the preprocessing stage, and convert them into class index tensors during training. On the loss-function side, alignment with this encoding is maintained through operations such as one-hot expansion. Specifically, labels are loaded with cv2.IMREAD_GRAYSCALE during preprocessing; in the training loop, label tensors are explicitly cast to long type and expanded into one-hot representations for Dice, Tversky, and related losses. This design ensures that label semantics are preserved under all interpolation and data augmentation operations, while remaining fully consistent with multi-class loss and evaluation interfaces. Its advantages lie in reducing interpolation artifacts and class ambiguity at object boundaries, thereby improving optimization stability and metric consistency.

#### 3.5.3. Synchronized Geometric Augmentation

To enhance the model’s robustness to variations in object orientation and imaging viewpoint, we apply synchronized geometric augmentations to both images and labels during training. These include horizontal/vertical flips and random rotations of ±5°. Bilinear interpolation is used for images, while nearest-neighbor interpolation with a fill value of 0 (background) is applied to labels. A shared random seed is employed to ensure strict alignment of the geometric transformations between images and labels. In practice, separate augmentation pipelines (img_aug and lbl_aug) are constructed, with InterpolationMode.NEAREST, fill = 0 explicitly set for label rotations. At sampling time, a random seed is first generated and then reused across both image and label augmentations to achieve synchronization. This design establishes the necessary invariance to orientation and viewpoint while preserving the discrete semantics of label boundaries. It can reduce overfitting, prevent class leakage caused by interpolation, and improve boundary quality as well as training stability.

#### 3.5.4. Lighting and Color Perturbations

To improve generalization across seasonal, temporal, and weather variations, we apply mild color jittering to the input images (brightness/contrast/saturation/hue magnitudes of 0.1/0.1/0.1/0.05, respectively). We incorporate ColorJitter into the image augmentation pipeline and use it in combination with geometric augmentations. This strategy not only simulates fluctuations in imaging conditions and expands the coverage of the color domain but also reduces the model’s sensitivity to exposure shifts, shadows, and color casts (e.g., yellowish or bluish tones), thereby enhancing robustness across spatiotemporal scenarios.

### 3.6. Deterministic Sliding-Window and Calibrated Pixel-Area Estimation

During inference, a deterministic sliding-window coverage is applied over the entire remote sensing image. The input image is read with OpenCV (BGR), and each 224 × 224 window undergoes the same normalization and tensor conversion (transform) used during training, before being fed into the segmentation network to produce per-pixel class scores. The class map is decoded directly from the logits using argmax and written into the global output buffer (out_map). When the image dimensions are not exact multiples of 224, the code generates the final row/column windows in an edge-aligned manner, where the last window deterministically overwrites overlapping regions. No weighted fusion or smoothing is applied, ensuring that images of arbitrary size are fully covered and that output remains unaffected by post-processing hyperparameters. Processing each window independently may introduce seam-like discontinuities at tile boundaries, especially when objects span across adjacent windows. In particular, (i) truncated context near patch borders can lead to boundary shifts and jagged edges for thin structures, (ii) overlapping regions may receive inconsistent logits from different windows, and the deterministic overwrite may select the less accurate prediction, and (iii) small false positives/negatives can concentrate around seams, which is more visible for narrow green strips, roads, or shadow-transition boundaries. Overlap-aware fusion (e.g., weighted averaging of logits) and padding-based strategies can mitigate such artifacts; however, we intentionally keep inference parameter-free to preserve auditability and to avoid introducing post-processing hyperparameters that could confound area estimation and reproducibility. Once the full-scene mask is generated, it is stored as a 2D integer array of class indices with the same dimensions as the ground-truth labels, where the Green class is consistently assigned to index 1. The area calculation starts from pixel counting. The number of pixels predicted as class index 1 in the full-scene output is denoted as $N_{pred}$, while the corresponding count in the ground-truth labels is denoted as $N_{gt}$. To quantify the relative error, we define the pixel-difference-based area accuracy as in Equation (9):

(9) $${AreaAcc}_{pixel}=(1-\frac{\left|N_{pred}-N_{gt}\right|}{max(1,N_{gt})})\times 100\%$$

Its essence is a percentual form of the Relative Absolute Error (RAE). Unlike common boundary-overlap metrics, this measure directly reflects the deviation in the “total amount of green area” and is insensitive to scattered boundary noise, making it better aligned with the decision-making needs of planning evaluation and ecological accounting. The conversion from pixel counts to physical area relies on an explicit scale file. Two random points are sampled on the satellite map; their pixel distance is denoted $D_{pixel}$, and their true distance is denoted $D_{real}$. The code parses these two scalars line by line and, based on them, computes the per-pixel area ($m^{2}/px$) as in Equation (10):

(10) $$u_{px}={\left(\frac{1000\times \;D_{real}}{D_{pixel}}\right)}^{2}$$

This formulation converts the measured length in kilometers ($D_{real}$, multiplied by ×1000 to obtain meters) and its corresponding pixel distance ($D_{pixel}$) into a linear scale in meters per pixel. Squaring this value yields the areal scale in square meters per pixel, thereby avoiding uncertain reliance on GDAL (Geospatial Data Abstraction Library) raster metadata or projection information. Accordingly, the physical areas of the prediction and the ground truth are, respectively, given by Equation (11):

(11) $$A_{pred}=N_{pred}\times u_{px}\;,\;A_{gt}=N_{gt}\times u_{px}$$

We aim to formalize green-area estimation into an auditable pipeline with minimal engineering assumptions, structured as semantic mask → pixel counting → calibrated conversion. Compared to approaches that rely on connected-component extraction, morphological refinements, or complex post-processing, our workflow introduces no posterior hyperparameters. Sliding-window coverage, class indexing, and counting rules are all explicitly fixed in code, making experiments easily reproducible and results directly comparable across datasets. The pixel-difference-based accuracy metric emphasizes “total quantity” rather than “boundary shape”, making it suitable for applications such as greening coverage monitoring, urban renewal assessment, and ecological red-line verification. In addition, the method offers three key advantages:

- Decoupling: Segmentation quality is independently evaluated using standard semantic metrics (e.g., Dice/mIoU, computed in code but excluded from area conversion), while scale error is separately controlled via the scale file containing $D_{pixel}$ and $D_{real}$. This separation facilitates diagnosis and correction.
- Robustness: A counting-based estimator is insensitive to local boundary jitter and isolated noise, providing a more stable reflection of overall green-space volume. Edge-aligned, deterministic sliding-window coverage eliminates the risk of gaps or double-counting caused by size rounding.
- Portability: The conversion requires only two scalar inputs to map pixel counts to physical area, allowing the same segmentation model to be reused across different platforms and datasets with varying ground resolutions, without rewriting inference or statistical logic.

### 3.7. Considerations for the Selection of Green Area Calculation Method

In this study, we adopt a deep learning-based semantic segmentation pipeline, rather than HSV color–space conversion with thresholding, for the specific purpose of quantifying urban green area from high-resolution imagery. This choice is dictated by the requirements of our implementation: the task is inherently multi-class-buildings, roads, water, background, and the target green class. Previous studies have shown that deep learning models have demonstrated superior performance in multi-class object segmentation tasks [36]. In addition, our downstream area estimator operates by pixel counting on the full-resolution semantic mask, followed by a deterministic physical-area conversion. Under these conditions, threshold-based HSV workflows are known to be sensitive to illumination changes, white-balance shifts, cast shadows, and local material variability, which undermines threshold portability across scenes and seasons; moreover, extending a single-threshold pipeline to reliable multi-class delineation typically introduces cascading rules and hand-tuned heuristics that conflict with the reproducibility required by pixel-accurate area auditing. In contrast, modern segmentation networks learn hierarchical, scene-aware features that jointly disambiguate vegetation from spectrally similar surfaces (e.g., painted roofs or artificial turf) while preserving fine boundaries needed by pixel-level aggregation. This learned representation integrates seamlessly with our sliding-window, no-postprocessing inference and the count-then-convert area estimator, yielding a method that is robust to photometric variability, naturally scalable to multiple land-cover types, and transparent for auditing: class indices are fixed, masks are produced deterministically by argmax, and green-area estimates derive directly from counted pixels without ad_hoc threshold tuning. Finally, because our codebase evaluates the same FPN decoder head across different backbones—including the Swin and SegFormer encoders used in the comparative experiments (detailed in Section 4)—the advantages of this segmentation-based paradigm are shown to hold consistently across architectures, thereby addressing potential concerns regarding scalability and generalizability.

## 4. Experiments

### 4.1. Dataset Description

The dataset used in this study was obtained from Satellites.Pro [37], consisting of 20 high-resolution remote sensing images selected from diverse urban regions worldwide. Each image covers an area that contains representative urban elements, including high-density buildings, roads, water bodies, and green spaces distributed either in patches or as continuous formations. To balance dataset scale with scene diversity, we generated training samples using a sliding-window cropping strategy (a window size of 224 × 224 with a stride of 112, corresponding to a 50% overlap), resulting in a total of 62,650 samples across the 20 images. Each image is accompanied by a per-image two-point scale record from Satellites.Pro, where the pixel distance $d_{P}$ corresponds to a real-world distance $d_{k}$ (km). We compute the ground sampling distance (GSD) as GSD = $d_{k}$ × 1000/$d_{P}$ (m/pixel), and the per-pixel area as $GSD^{2}$ ($m^{2}$/pixel). Across the test images, the GSD ranges from 1.39 to 1.84 m/pixel (mean ± std: 1.64 ± 0.23 m/pixel). All samples are organized in a CSV index file with one-to-one mappings between image and label entries, ensuring reproducible data loading and partitioning. All original satellite maps were annotated using LabelMe for multi-class semantic segmentation, with five classes defined as follows: {0} background, {1} green space, {2} building, {3} road, and {4} water. Label definitions and example annotations are provided in Appendix A (Table A1). Green space was explicitly assigned to label value {1} to support high-precision segmentation and area quantification, while buildings, roads, and water classes were included to explicitly capture common occlusion, adjacency, and boundary overlap in complex urban scenes, thereby enabling more reliable boundaries and contextual understanding.

### 4.2. Details of Training and Implementation

Unless otherwise specified, to ensure fair and consistent comparability, GreenViT and all baseline models in the comparative experiments are fully aligned in terms of optimizer, learning-rate scheduling, data augmentation pipeline, input specifications, evaluation frequency, and early-stopping criteria. All models are trained under a unified training protocol with an input size of 224 × 224, a batch size of 16, and five-fold cross-validation (K = 5). We employ the AdamW optimizer with a CosineAnnealingLR scheduler, where T_max is set to 20,000, and the weight decay is 1 × 10^−2^. Model parameters are divided into two groups: the encoder (pretrained backbone) and the decoder head. A consistent dual learning-rate strategy is applied across all models, with a backbone learning rate of 5 × 10^−6^ and a head learning rate of 1 × 10^−3^. This design is motivated by the need to mitigate the potential prior advantage of large-scale pretrained encoders during the early optimization stage and to prevent convergence imbalance caused by differences in model capacity. During both training and validation, all models use the same composite loss function, consisting of a fixed-weight combination of Cross-Entropy, Dice, Focal, and Tversky losses, which is particularly effective under class-imbalanced segmentation scenarios. Validation is performed every 1000 training steps using identical evaluation metrics for all models, and early stopping is triggered when the mIoU does not improve for 20 consecutive validations (PATIENCE = 20). For evaluation, we strictly follow a unified sliding-window inference protocol, along with standardized measurements of inference latency and memory usage, to ensure fair and reproducible comparisons across models. In addition, our experiments were performed on a workstation equipped with an NVIDIA GeForce RTX 3090 GPU, 128 GB RAM, and Windows 11. The primary software environment included Python 3.9, PyTorch 2.3.1, CUDA 11.8, and OpenCV 4.10.0.

### 4.3. Model Architectures

**Figure 3:**
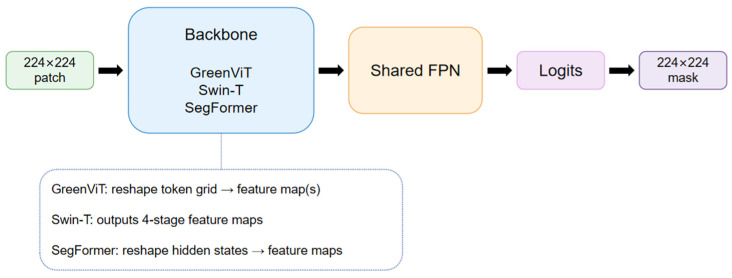
General workflow of the comparative models under a unified protocol. A 224 × 224 patch is processed by a Transformer backbone (GreenViT/Swin-T/SegFormer-B0) and decoded by a common head (FPN or the proposed Green Head) to produce a 5-class segmentation mask.

We conducted experiments with four model configurations to evaluate segmentation accuracy and efficiency under a unified protocol. All comparative baselines follow the same high-level workflow (Figure 3):

A 224 × 224 patch is fed into a transformer backbone, backbone features are passed to a decoder head (either the shared FPN head or the proposed Green Head), and the output logits are resized to the original patch resolution to obtain a 5-class prediction mask. (a) GreenViT + Green Head: This is our proposed model, built on a ViT-L/14 encoder combined with the single-path progressive upsampling decoder (Green Head). We employ an ImageNet-pretrained ViT-L/14 backbone to provide strong global context modeling. (b) Swin-T + FPN: Swin-T is a hierarchical Transformer with shifted-window self-attention. We use the official ImageNet-1k pretrained Swin-T weights (timm). The backbone outputs multi-stage feature maps, which are fused by a unified FPN head to produce segmentation logits. (c) SegFormer-B0 (HF) + FPN: SegFormer employs a lightweight MiT-B0 encoder. We use the official ImageNet-1k pretrained weights (Hugging Face). Hidden sequences from four stages are reshaped into 2D feature maps and fused by the same unified FPN head as in (b). (d) GreenViT + FPN: To isolate the effect of the proposed Green Head, we also evaluate GreenViT with the same FPN head used in the baseline models. This configuration uses the same ViT backbone but replaces the decoder with the shared FPN for controlled comparison.

We chose ViT-L/14 as the representative pure-ViT backbone for three practical reasons. First, with a 224 × 224 input, a 14 × 14 patch size yields an exact 16 × 16 token grid, which matches the design of our single-path decoder and enables a clean progressive upsampling schedule to recover full resolution without introducing extra resizing heuristics. Second, the ViT-L capacity provides strong global-context modeling that is beneficial for boundary-rich urban mosaics, while ImageNet pretraining offers strong regularization under a limited number of source scenes. Third, compared with smaller-patch variants (e.g., /8), the /14 tokenization reduces the token count and thus attention cost, making training and inference feasible on a single GPU, while still maintaining finer granularity than coarser patching.

The encoder of the Swin-T + FPN model is implemented using timm’s swin_tiny_patch4_window7_224 with features_only = True, which outputs the feature pyramid across different layers before feeding into a unified FPN head. After instantiation, the code performs a “channel probing” step, where a dummy input is forwarded once to dynamically record the actual channel dimensions (actual_channels) of each stage, ensuring automatic alignment across different implementations or pretrained weights. The detected channel tuple is then used to initialize the FPNHead. During forward propagation, the backbone produces multi-level features (backbone(x)), which are subsequently passed to the FPN to generate semantic segmentation logits, consistent with the other models. Within the decoder, FPN applies a 1 × 1 convolution to project each stage’s feature map to a unified dimensionality of fpn_dim = 256, followed by BatchNorm and ReLU activation. These features are then bilinearly upsampled to a common spatial resolution, concatenated along the channel dimension, fused with a 3 × 3 convolution, and passed through a final 1 × 1 classification convolution to obtain the prediction, which is further interpolated back to the input resolution. The encoder of SegFormer-B0 (from Hugging Face) is configured with output_hidden_states = true to return the hidden sequences of four stages. To interface with FPN, each sequence of shape [B, L, C] is reshaped into a 2D feature map of shape [B, C, P, P], where P is derived from $\sqrt{L}$, thereby forming a multi-scale feature list. For consistent comparison, the implementation fixes the MiT-B0 channel configuration (32, 64, 160, 256) when initializing the unified FPN head, while maintaining the same forward interface as the Swin-T pathway (feats → FPNHead → logits). The decoder is identical to that of Swin-T + FPN and GreenViT + FPN in the comparative experiments, with a unified implementation and shared hyperparameters.

#### Rationale for Selecting Swin-T and SegFormer-B0 (HF) as Baseline Models and FPN as the Comparative Decoder Head

We chose Swin-T and SegFormer-B0 (HF) as baseline models primarily because they are highly representative and collectively cover two mainstream branches of Vision Transformers. Swin-T exemplifies the hierarchical, window-based self-attention family, whereas SegFormer-B0 (MiT-B0) represents the lightweight pyramid-style design. Both models are widely adopted in academic and industrial practice, with well-established reproduction pipelines, and are instantiated in our experiments using official pretrained weights and interfaces from timm and transformers, ensuring stability and reliability. A further reason for this choice is their moderate pretraining scale, which contributes to fairness by avoiding the confounding effects of overly strong pretraining. Since all models are trained and evaluated with the same script and decoder, the comparison emphasizes encoder inductive biases and the innovations of our proposed decoder. In addition, both architectures are naturally compatible with FPN: Swin-T natively outputs multi-level [B, C, H, W] feature maps, while SegFormer-B0 produces an equivalent four-stage pyramid via sequence-to-feature-map reshaping in our codebase. This seamless integration ensures that the decoder remains a controlled constant, thereby isolating performance differences to the encoder and our new decoder design. FPN itself was selected as the comparative decoder because of its simplicity, robustness, and backbone-agnostic nature; it relies only on lightweight 1 × 1 projections, BatchNorm with ReLU, bilinear upsampling, and a single 3 × 3 fusion convolution, and can thus flexibly accommodate Swin-T, MiT-B0, or ViT-based features. Its align-then-fuse paradigm, which unifies channel dimensionality and spatial resolution before feature fusion, provides a clean and consistent interface across different backbones. Finally, its lightweight nature avoids introducing excessive parameters or operations, which not only facilitates clear efficiency–accuracy analysis under normalized metrics but also prevents the decoder from overshadowing the contributions of the encoder or our proposed Green Head.

### 4.4. Metrics

To comprehensively evaluate the performance of each model, we employed the following standard metrics: Dice coefficient, which measures the overlap between predictions and ground-truth annotations, with higher values indicating better segmentation quality; mIoU (macro), which computes the average IoU across all classes and serves as a key indicator of segmentation accuracy; Pixel Accuracy (PA), which quantifies the proportion of correctly classified pixels and reflects the overall classification performance of the model; and Per-class IoU, which reports the IoU for each individual class, enabling a more fine-grained analysis of segmentation performance across categories. In addition to these conventional metrics, we also considered resource and efficiency-related factors automatically recorded from the training logs, including the number of computational cost (FLOPs), peak memory consumption (PeakMem, MB), and throughput (FPS). Based on these, we further report the following normalized indicators for each model and each fold:

- Compute-normalized accuracy:
  (12) $$mIoU/GFLOP=\frac{mIoU}{\max(10^{-6},FLOPs(G))}$$
- Throughput-weighted accuracy:
  (13) mIoU×FPS
- Memory-normalized accuracy:
  (14) $$mIoU/\mathrm{GB}=\frac{mIoU}{\max(10^{-6},PeakMem(\mathrm{MB})/1024)}$$
- Primary class (Green) indicators:
  (15) $$IoU_{Green}/GFLOP,IoU_{Green}\times FPS$$

The purpose of normalized evaluation is to assess the relative gains per unit cost, rather than to substitute for the absolute task accuracy.

### 4.5. Main Results (Five-Fold) and Visualization of Comparative Results

Unless otherwise specified, the data in each table corresponds to the best-performing value within the respective fold. Appendix A (Table A2) presents the log outputs of Loss, Dice, mIoU, PA, mPA, Focal, and Tversky metrics for the four models under five-fold cross-validation. We can observe that the combination of our proposed GreenViT and Green Head consistently outperforms the baselines across all metrics in folds F1–F5. Appendix A (Table A3, Table A4, Table A5 and Table A6) presents the per-fold results (normalized indicators) for each model, with the per-class Green metrics reported in the last two columns. Averaging over the five folds yields the fold-wise consistency and overall summary, as presented in Table 1. All averages are computed from logs recorded under the unified training loop.

**Figure 4:**
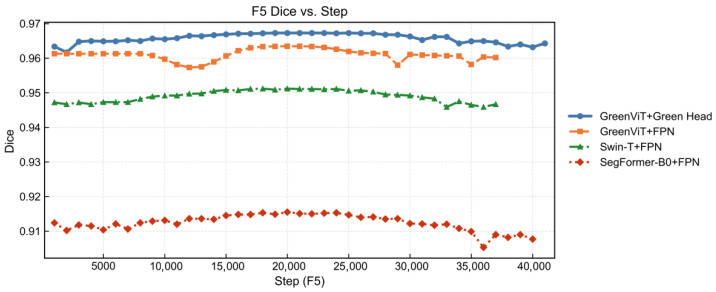
Comparison of Dice scores.

**Figure 5:**
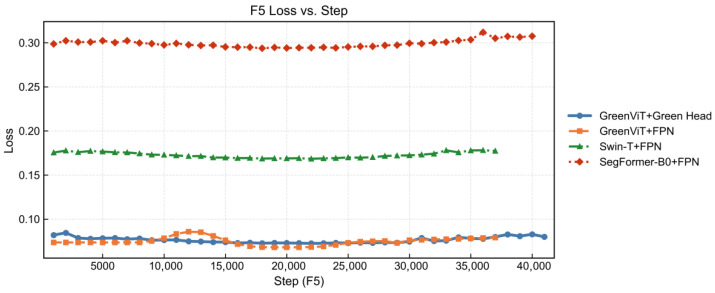
Comparison of loss.

To more intuitively demonstrate the effectiveness, convergence, and stability of our model, we further constructed comparative visualizations based on three metrics_Dice, Loss during the fifth fold (F5) validation phase, as shown in Figure 4 and Figure 5.

The combination of GreenViT and Green Head continues to perform strongly in the fifth fold validation, with its Dice coefficient consistently surpassing the other three model combinations throughout the entire validation phase, reaching a maximum value of 0.9671. The combination of GreenViT and FPN also delivers promising results, although it still falls short of the GreenViT + Green Head, it achieves a maximum Dice coefficient of 0.9635. By contrast, the highest Dice coefficient for Swin-T + FPN is 0.9512, while SegFormer-B0 + FPN achieves only 0.9155. The Loss curves further highlight the superior convergence ability of the GreenViT + Green Head combination. Throughout the fifth fold validation, its Loss remains consistently below 0.1, with stable performance from the beginning of validation until early stopping, without notable oscillations. Although the GreenViT + FPN combination shows slight increases in Loss around 10,000 and 15,000 steps, it quickly reconverges and stabilizes. In contrast, both the Swin-T + FPN and SegFormer-B0 + FPN combinations, while relatively stable without large fluctuations, maintain Loss values above 0.15; notably, SegFormer-B0 + FPN exceeds 0.3 in the later validation phase, underscoring its gap relative to our GreenViT. The Dice curves mainly reflect region overlaps, whereas the composite loss is additionally sensitive to hard pixels and minority-class confusion (via Focal/Tversky terms). Therefore, a model can exhibit comparable Dice but still maintain a higher loss if residual errors concentrate on boundaries, thin structures, or low-frequency classes. In our setting, the smoother and lower loss profile of GreenViT + Green Head suggests more stable handling of hard boundary pixels and fewer oscillations in class-wise trade-offs during validation. The occasional loss bumps observed for GreenViT + FPN likely indicate higher sensitivity to ambiguous boundary regions (e.g., seam-adjacent pixels or shadow-transition areas) before reconvergence, while the persistently higher loss of Swin-T/SegFormer indicates systematic residual confusion that does not vanish with longer validation steps under the same protocol.

### 4.6. Analysis and Discussion of Results

**Figure 6:**
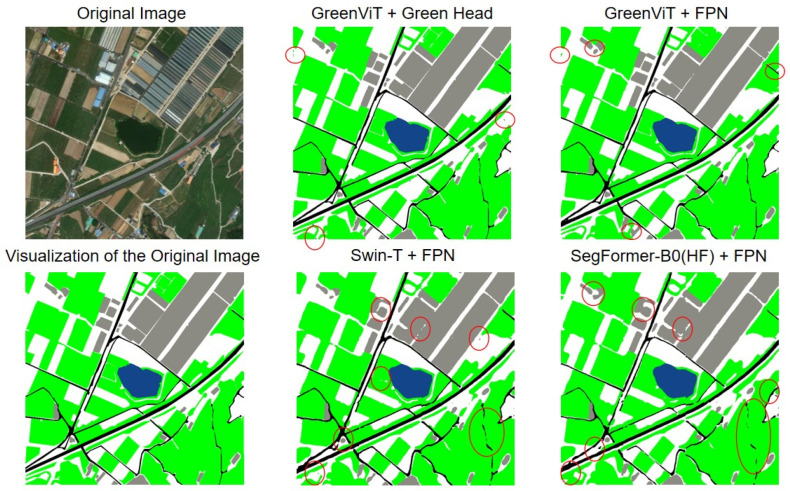
Comparison of actual output visualizations (the areas with larger differences are marked with red circles).

As expected, the two lightweight backbones—SegFormer-B0 (HF) and Swin-T—demonstrate substantially higher efficiency in terms of compute- and memory-normalized metrics. For instance, SegFormer-B0 achieves an average of 0.0516 mIoU/GFLOPs and 4.93 mIoU/GB across the five folds. However, in terms of absolute accuracy, our GreenViT + Green Head consistently achieves the best performance, with a five-fold average of mIoU = 0.9200 and IoU_Green = 0.9594. This trade-off is expected and is clearly captured and quantified by our normalized indicators. However, due to differences in pretraining scale and architectural design, we do not claim that GreenViT inevitably outperforms all methods under any capacity. Instead, our conclusion is more constrained: under a unified training protocol and within the current computational budget, GreenViT achieves higher and more stable accuracy in five-fold evaluations, while also demonstrating a favorable accuracy–efficiency trade-off from a capacity-aware perspective. Although the inference memory footprint of GreenViT is larger than that of its lightweight counterparts, this cost translates into substantially higher segmentation quality, particularly in boundary details across all categories. As illustrated in Figure 6 (we highlight representative regions with notable differences using red circles), where we compare the visualization outputs of four models, the combination of GreenViT and the proposed Green Head delivers excellent results in the categories of Green, Building, Road, Water, and Others. While competing models perform reasonably well on the Green class, their segmentation and prediction quality for other categories are markedly inferior. The combination of GreenViT and FPN also demonstrates strong performance; nevertheless, it still lags behind our GreenViT + Green Head, further underscoring the strength of the GreenViT architecture and the Green Head design. Consequently, GreenViT is particularly well-suited for deployment on the server side or in offline mapping scenarios, as well as applications such as urban planning and green-space statistics, where accuracy is critical. For deployment constrained by edge devices, we recommend using a lightweight configuration of our model or adopting smaller input resolutions. Even under such conditions, normalized indicators such as mIoU/FLOPs and mIoU/Params show that GreenViT maintains a strong balance between accuracy and efficiency.

#### Quantitative Accuracy of Area Estimation and Per-Class Results

For the green area quantification evaluation, we compute test-Dice, the pixel-level area agreement (AreaAcc(pixel)), and absolute area estimates using a per-pixel area file. At the best checkpoints, relative area error is 1.10% (GreenViT), 3.77% (Swin-T), and 6.73% (SegFormer-B0), indicating that higher segmentation quality translates into more faithful area quantification, which is crucial for downstream urban green-space measurement. Our method outperforms the other models in handling fine-grained details and continues to demonstrate strong segmentation capability even under more complex conditions with richer image content. Not only does it perform well on green spaces, but it also achieves high accuracy in the three additional categories of Building, Water, and Road, as summarized in Table 2 (mean ± standard deviation over five folds), with the full per-fold per-class results provided in Table A7.

Our GreenViT, combined with the Green Head, consistently outperforms the other three model groups across all folds. Notably, even for challenging categories such as buildings—with diverse morphologies and heavy shadow occlusions—our model achieves an IoU as high as 91%, while for roads and water it reaches 93.3% and 95.3%, respectively. These results support our belief that beyond green-space segmentation, our model holds strong potential to make valuable contributions to multiple domains of urban planning in the future.

### 4.7. Cross-Dataset Validation

**Figure 7:**
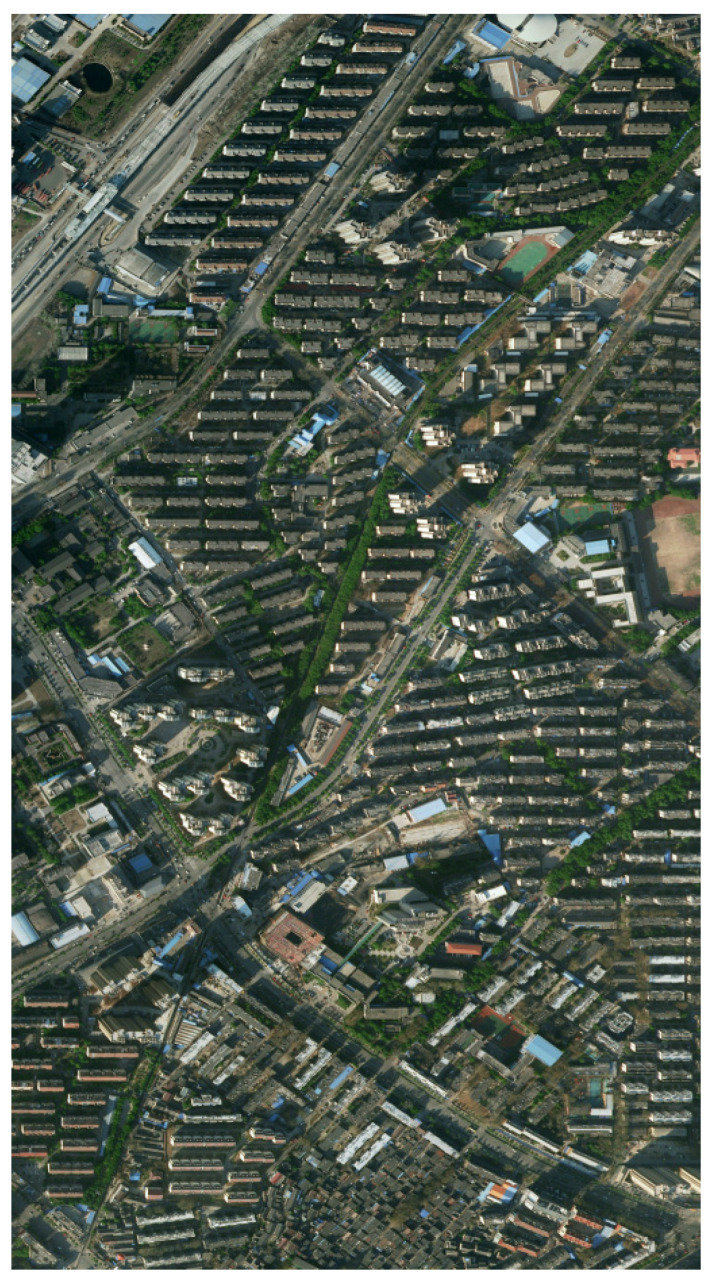
Sample image from the CCF.

To assess the generalization ability of our model framework, cross-dataset validation was performed using an external dataset named the CCF (China Computer Federation) Satellite Imagery AI Classification and Recognition Dataset [38], comprising high-resolution satellite images from geographically diverse urban regions. This dataset included images with varying land cover characteristics, lighting conditions, and spatial resolutions. As shown in Figure 7, this is a sample image from the CCF, characterized by high complexity, significant shadow occlusions, and rich details.

**Figure 8:**
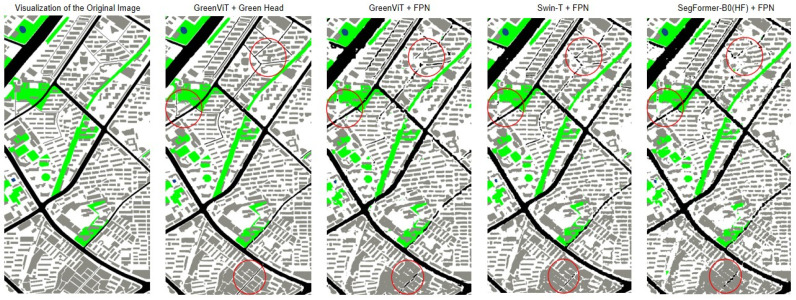
Comparison of cross-dataset segmentation results for different models(the areas with larger differences are marked with red circles).

To ensure a fair comparison, we conducted the cross-dataset validation experiments under the same configuration as in the comparative study, and we selected, for each model, the visualization output corresponding to the highest mIoU achieved in the fifth fold for comparison, as shown in Figure 8 (we highlight three representative regions with notable differences using red circles).

### 4.8. Ablation Study

#### 4.8.1. Motivation and Design

Ablation matrices: (1) Removal: turn off one component at a time (e.g., −Dice, −Focal, −Tversky, head→FPN). (2) Replacement: swap decoder (GreenViT ↔ FPN); loss baselines (CE-only; CE + Dice). (3) Incremental: A, B, C, A + B, A + C, B + C, A + B+C, where A: GreenViT+ Green Head (vs FPN), B: Composite loss (vs CE-only), C: Full Augmentation (vs no augmentation). (4) Robustness: repeat key rows under three backbones (ViT tokens + FPN, Swin-T+FPN, SegFormer-B0+FPN). Each configuration was evaluated with at least three random seeds (e.g., 0/1/2), and results are reported as mean ± standard deviation. Statistical significance was assessed using paired *t*-tests or Wilcoxon signed-rank tests when normality assumptions were violated, against the corresponding baseline. Significant differences are denoted as * p < 0.05 and ** p < 0.01 in the tables. To ensure robustness, an observed improvement was considered reliable only if it exceeded the standard deviation of the corresponding configuration.

#### 4.8.2. Ablation Study Results and Analysis

To ensure a clear progression of configurations, we adopt GreenViT + FPN + CE-only + no-aug as the minimal baseline. We then incrementally incorporate B (replacing CE-only with the Composite Loss) and C (enabling Full Augmentation), and finally add A (replacing the FPN with the Green Head). The ablation studies’ results are reported in Table 3, Table 4 and Table 5.

Finally, we obtained: +A (n = 5 folds): mIoU t = 5.71,  p < 0.01, Dice t = 5.54, p < 0.01 (paired *t*-test with fold-wise pairing). Starting from the CE-only + no-aug baseline (mIoU 0.883, Dice 0.937), introducing the composite loss (B) improves performance by + 0.014 mIoU (+1.6%) and + 0.009 Dice (+1.0%) to 0.897/0.946. Enabling Full Augmentation (C) yields a further + 0.009 mIoU (+1.0%) and + 0.004 Dice (+0.4%), reaching 0.906/0.950. Replacing the FPN with Green Head yields an additional +0.014 mIoU (from 0.9060 to 0.9200), i.e., +1.5% relative to the preceding configuration, and increases Dice to 0.9580 ± 0.0135 (p < 0.01, paired t-test). These gains are statistically significant relative to the previous configuration (p < 0.01, paired test as marked). Pixel-wise metrics align with these improvements (PA 0.9570, mPA 0.9588). Collectively, all three levers contribute positively: the decoder swap (A) provides the single largest measured step on top of a strong training recipe, while loss composition (B) primarily addresses class imbalance and hard pixels, and augmentation (C) improves boundary fidelity and photometric robustness, yielding steady, additive gains from the minimal baseline.

Using GreenViT + FPN + Composite + Full aug as the common baseline, swapping the decoder to Green Head increases mIoU to 0.9200 ± 0.0243 and Dice to 0.9580 ± 0.0135, confirming that the decoder remains a primary performance driver even with a well-tuned training recipe. In contrast, replacing the backbone while keeping the shared FPN head degrades accuracy: Swin-T + FPN yields −0.0237 mIoU (−2.62%) and −0.0135 Dice (−1.43%) relative to the baseline, whereas SegFormer-B0(HF) + FPN incurs larger drops of −0.0925 mIoU (−10.21%) and −0.0546 Dice (−5.75%). The consistent ranking—GreenViT+ Green Head > GreenViT+FPN > Swin-T+FPN > SegFormer-B0+FPN across mIoU/Dice/PA—indicates that while backbone capacity governs absolute accuracy, the decoder choice still provides a tangible, orthogonal boost.

Beyond accuracy, Green Head is structurally simpler in the pure-ViT setting. It consumes a single 16 × 16 token feature grid and applies four sequential upsampling–refinement stages, resulting in a single-stream data flow. In contrast, FPN requires assembling and storing a multi-level feature pyramid, performing lateral projections per level, and fusing features across branches before prediction. This increases the number of feature tensors and fusion nodes that must be managed and tuned. Therefore, Green Head reduces branching and integration points, simplifies implementation and debugging, and improves reproducibility while still delivering consistent accuracy gains over FPN under the same backbone and protocol. Under identical settings, GreenViT + Green Head delivers the best aggregate performance (mIoU 0.9200 ± 0.0243, Dice 0.9580 ± 0.0135, PA 0.9570 ± 0.0149). Holding the backbone fixed, replacing Green Head → FPN lowers mIoU to 0.9059 ± 0.0272 and Dice to 0.9501 ± 0.0153, indicating that the observed gains are attributable to the decoder rather than encoder confounds. Per-class IoU on GreenViT further corroborates this effect: Green Head outperforms FPN on Building (89.06% vs. 87.46%), Road (91.48% vs. 89.68%), Water (94.26% vs. 93.44%), and Green (95.98% vs. 95.24%), with the largest gains on Road and Building—categories characterized by thin structures and complex boundaries—consistent with the qualitative advantage of a single-path upsampling head. In conclusion, the decoder emerges as the most influential factor at convergence (+1.5% mIoU and +0.8% Dice over an already strong baseline). Improvements from loss composition (B) and augmentation (C) accumulate steadily from the minimal baseline, and the decoder (A) adds further gains on top—indicating additive rather than redundant effects along the incremental path. While absolute accuracy tracks backbone capacity, the decoder’s relative advantage remains stable across backbones, supporting the portability of this finding. Per-class analyses show that the decoder swap particularly benefits thin/elongated, boundary-rich categories (e.g., Road, Building), while still yielding smaller but consistent gains on more compact classes (Water, Green). Accordingly, the recommended operating point is Green Head + Composite loss + Full Augmentation on GreenViT, which achieves the highest aggregate metrics across all tables.

## 5. Conclusions, Limitations, and Future Work

### 5.1. Conclusions

This work addresses the need for reliable, scalable monitoring of urban green space in support of climate-adaptation and pollution-control governance. We proposed GreenViT, a ViT-based segmentation framework with a single-path, progressive upsampling head named Green Head, a fixed-weight composite loss, and a pixel-to-physics calibration pipeline for area estimation. The decoder restores resolution through bilinear upsampling at successive scales with lightweight 1 × 1/3 × 3 refinements, purposely avoiding multi-branch fusion or transposed convolutions to preserve the ViT’s global context while sharpening boundaries. The loss couples Cross-Entropy, Dice, Focal (γ = 2), and Tversky (α = 0.7, β = 0.3) to address class imbalance and contour fidelity. Together with a deterministic sliding-window inference and post_hoc conversion from pixel counts to physical area via an explicit scale file, the system forms an auditable end-to-end pipeline targeting both high-precision segmentation and reliable quantification. Across five-fold evaluations, GreenViT + Green Head consistently outperformed the baselines (GreenViT + FPN, Swin-T + FPN, SegFormer-B0 + FPN). The best aggregate performance was mIoU = 0.9200 ± 0.0243 with Dice = 0.9580 ± 0.0135 and PA = 0.9570 ± 0.0149, exceeding the alternatives under a unified protocol. The analysis further quantified the expected trade-off: lightweight backbones (e.g., SegFormer-B0, Swin-T) offered stronger compute/memory normalization, while GreenViT + Green Head achieved the highest absolute accuracy and favorable accuracy–efficiency balance. For the quantification task, converting predicted masks to physical area yielded relative area error of 1.10% (GreenViT) versus 3.77% (Swin-T) and 6.73% (SegFormer-B0) at best checkpoints, indicating that segmentation quality translates into more faithful area measurement. The calibration is transparent—pixel counting followed by a two-point scale conversion—and intentionally decoupled from semantic metrics to simplify diagnosis of segmentation versus scaling errors. Ablation studies corroborated the contributions: starting from a minimal baseline (GreenViT + FPN, CE-only, no augmentation), introducing the composite loss and synchronized geometric + color augmentations improved mIoU/Dice incrementally, and swapping FPN for the proposed Green Head delivered the largest additional gain, with differences marked * p< 0.01 under paired testing.

The end-to-end chain—mask → pixel counting → calibrated conversion—is transparent and reproducible, decoupling semantic accuracy from scale calibration for diagnostics and auditability. manuscript In practice, the resulting policy-ready indicators support planning evaluation, green-space statistics, urban-renewal assessment, and ecological red-line verification, enabling long-term monitoring aimed at heat-mitigation and air-quality co-benefits in dense cityscapes. In summary, the current study is limited by the number of source scenes and the fixed five-class taxonomy, and the highest-accuracy configuration (ViT-L/14) has non-trivial memory requirements for large-scale deployment. Future work will therefore (i) expand to larger and more diverse benchmarks with stricter cross-scene evaluation, (ii) establish quantitative cross-dataset tests beyond qualitative visualization, and (iii) explore more resource-aware variants (smaller backbones, higher-resolution training for tiny greens, and overlap-aware inference) to better balance accuracy and deployment cost.

### 5.2. Limitations

First, dataset scale and composition, experiments used 20 high-resolution urban scenes (62,650 patches via sliding window) spanning five classes: background, green, building, road, water. While scenes were geographically diverse, the evaluation is bounded by this taxonomy and by the number of source images. Second, deployment constraints and hardware requirements: due to the high-capacity ViT backbone, GreenViT has a larger memory footprint than lightweight alternatives, and attention-based backbones generally scale in memory with the token grid size. Although our 224 × 224 patch-based inference (and the sliding-window strategy for full-scene coverage) keeps per-window computation bounded, running large-scale mapping efficiently still benefits from modern GPUs and sufficient memory bandwidth. In our experiments, training and profiling were conducted on a single NVIDIA GeForce RTX 3090 workstation; on smaller GPUs or edge devices, practical deployment may require reduced batch sizes, gradient accumulation, smaller backbones, or lower input resolution, trading accuracy for resource feasibility. Third, pipeline assumptions: the quantification stage depends on an external per-image scale file derived from two-point measurements. Although this design enhances portability and avoids reliance on GIS raster metadata, area estimates are conditioned on the correctness of these calibration inputs. The inference scheme also uses deterministic, edge-aligned sliding windows without overlap blending or post-processing, prioritizing reproducibility and parameter-free operation. Fourth, generalization evidence: cross-dataset validation on the CCF dataset was presented through comparative visualizations prepared under the same configuration; while informative, this evaluation was primarily qualitative. Fifth, small/fragmented green areas: very small or highly fragmented green patches (e.g., tiny lawns, street-tree crowns, narrow median strips) may be under-segmented because tokenization and successive upsampling impose an implicit minimum resolvable scale, and such fragments contribute limited signal under strong class imbalance. In these cases, errors tend to appear as missed tiny greens or slightly eroded boundaries. Potential remedies include training at higher input resolution, adopting smaller patch sizes or hierarchical backbones, adding boundary-aware supervision, and using overlap-aware fusion during inference.

### 5.3. Future Work

Future work will focus on broadening the evaluation and taxonomy. Expanding beyond the current five-class setup and increasing the number and diversity of source scenes would enable stronger stress tests of generalization and class granularity relevant to urban applications. Quantitative cross-dataset benchmarks—complementing the provided qualitative CCF comparisons—are a direct next step under the established protocol. A second line will target resource-aware deployment. Given the observed accuracy–efficiency trade-offs and memory footprint, it is natural to develop and benchmark lightweight GreenViT configurations and reduced resolutions for edge devices, within the same training and inference controls reported here. Such experiments align with the stated recommendations and would clarify the attainable operating points under tighter compute and memory budgets. Third, calibration and auditing of area estimation can be strengthened. While the pixel-count-to-area conversion is deliberately simple and decoupled, formalizing procedures for collecting and validating per-image scale files across deployment contexts would further stabilize physical estimates and ease transfer across datasets with varying ground resolutions. Finally, methodological extensions suggested by the ablations include continued study of training recipes (e.g., composite-loss variants and augmentation schedules) under the same unified evaluation, as the experiments show additive benefits and statistically significant gains (italic *p* < 0.01) over the minimal baseline. Additional analyses can probe decoder–backbone interactions to systematically map where the single-path upsampling head yields the largest returns, especially on boundary-rich categories. Collectively, these directions follow directly from the presented evidence: the proposed architecture and training scheme deliver state-of-the-art accuracy within the study’s scope, the quantification pipeline is transparent and portable, and clear avenues exist to expand coverage, strengthen generalization, and tailor the system for constrained deployments.

## Figures and Tables

**Table 1:** Fold-wise consistency and summary averaged over 5 folds.

GreenViT + Green Head	
mIoU	0.9200 ± 0.0243
IoU_Green	0.9594 ± 0.0183
mIoU/GFLOPs	0.0052 ± 0.0001
mIoU/GB	0.3971 ± 0.0105
mIoU × FPS	0.0920 ± 0.0024
IoU_Green/GFLOPs	0.0054 ± 0.0001
IoU_Green × FPS	0.0959 ± 0.0018
GreenViT + FPN	
mIoU	0.9059 ± 0.0272
IoU_Green	0.9518 ± 0.0206
mIoU/GFLOPs	0.0038 ± 0.0001
mIoU/GB	0.3718 ± 0.0112
mIoU × FPS	0.0906 ± 0.0027
IoU_Green/GFLOPs	0.0039 ± 0.0001
IoU_Green × FPS	0.0952 ± 0.0021
Swin-T + FPN	
mIoU	0.8822 ± 0.0274
IoU_Green	0.9434 ± 0.0203
mIoU/GFLOPs	0.0370 ± 0.0012
mIoU/GB	3.4454 ± 0.1072
mIoU × FPS	0.1764 ± 0.0055
IoU_Green/GFLOPs	0.0396 ± 0.0009
IoU_Green × FPS	0.1887 ± 0.0041
SegFormer-B0(HF) + FPN	
mIoU	0.8135 ± 0.0327
IoU_Green	0.8972 ± 0.0329
mIoU/GFLOPs	0.0516 ± 0.0021
mIoU/GB	4.9318 ± 0.1982
mIoU × FPS	0.1627 ± 0.0065
IoU_Green/GFLOPs	0.0570 ± 0.0021
IoU_Green × FPS	0.1794 ± 0.0066

**Table 2:** Per-class segmentation performance (IoU, %) on the test set (mean ± standard deviation over five folds).

Model	Building IoU (%)	Road IoU (%)	Water IoU (%)	Green IoU (%)
GreenViT + Green Head	89.06 ± 2.81	91.48 ± 2.68	94.26 ± 1.40	95.98 ± 1.74
GreenViT + FPN	87.46 ± 3.20	89.68 ± 2.90	93.44 ± 1.62	95.24 ± 1.99
Swin-T + FPN	83.02 ± 3.06	87.24 ± 3.08	92.56 ± 1.74	94.38 ± 2.04
SegFormer-B0 (HF) + FPN	75.36 ± 3.55	79.82 ± 3.19	87.74 ± 2.45	89.76 ± 3.32

**Table 3:** Incremental ablation (A = Green Head, B = Composite Loss vs. CE-only, C = Full Augmentation). “↑” indicates that higher values are better. “→” indicates “replacement”. “**” indicates statistically significant improvement compared to the previous configuration (*p* < 0.01, paired *t*-test across five folds).

Setting	Baseline: GreenViT + FPN, CE-Only, No-Aug	+B (Composite Loss: C→ CE + Dice + Focal + Tversky)	+C (Full Aug: Sync-Geo + Color Jitter)	+A (Decoder Swap: FPN→ Green Head)
mIoU macro ↑	0.883 (±0.028)	0.897 (±0.027)	0.906 (±0.027)	0.9200 (±0.0243 **)
Δabs	-	+0.014	+0.009	+0.014
Δ%	-	+1.6%	+1.0%	+1.5%
Dice macro ↑	0.937 (±0.016)	0.946 (±0.015)	0.950 (±0.015)	0.9580 (±0.0135 **)
Δabs	-	+0.009	+0.004	+0.008
Δ%	-	+1.0%	+0.4%	+0.8%
PA ↑	0.936 (±0.018)	0.944	0.948	0.9570 (±0.0149)
mPA ↑	0.939 (±0.016)	0.947	0.951	0.9588 (±0.0135)

**Table 4:** One-factor ablation results with respect to a common baseline (GreenViT + FPN + Composite Loss + Full Augmentation). “↑” indicates that higher values represent better performance. “←” denotes replacing the FPN decoder with the Green Head while keeping all other settings fixed. “**” indicates statistically significant differences compared to the baseline (*p* < 0.01, paired *t*-test across five folds).

Component (On vs. Baseline)	Head: Green Head (←FPN)	Backbone: Swin-T + FPN	Backbone: SegFormer -B0(HF) + FPN	+Dice (on CE-Only)	+Focal (on CE+Dice)	+Tversky (α = 0.7, β = 0.3)
mIoU macro ↑	0.9200 ± 0.0243 **	0.8822 ± 0.0274 **	0.8135 ± 0.0327 **	+0.9%	+0.6%	+0.5%
Δabs	+0.0141	−0.0237	−0.0925	-	-	-
Δ%	+1.56%	−2.62%	−10.21%	-	-	-
Dice macro ↑	0.9580± 0.0135 **	0.9366 ± 0.0159 **	0.8955 ± 0.0204 **	+1.2%	+0.5%	+0.6%
Δabs	+0.0079	−0.0135	−0.0546	-	-	-
Δ%	+0.83%	−1.43%	−5.75%	-	-	-
PA ↑	0.9570 ±0.0149	0.9348 ± 0.0176	0.8916 ± 0.0225	-	-	-
mPA ↑	0.9588± 0.0135	0.9386 ± 0.0164	0.8972 ± 0.0202	-	-	-

**Table 5:** Robustness across backbones (resolution/domain fixed at default). “↑” indicates that higher values represent better performance.

Decoder	Green Head	FPN	FPN	FPN
Loss	Composite	Composite	Composite	Composite
Augmentation	Full	Full	Full	Full
Backbone	GreenViT	GreenViT	Swin-T	SegFormer -B0(HF)
Res	224	224	224	224
mIoU macro ↑	0.9200 ± 0.0243	0.9059 ± 0.0272	0.8822 ± 0.0274	0.8135 ± 0.0327
Dice macro ↑	0.9580 ± 0.0135	0.9501 ± 0.0153	0.9366 ± 0.0159	0.8955 ± 0.0204
PA ↑	0.9570 ± 0.0149	0.9483 ± 0.0168	0.9348 ± 0.0176	0.8916 ± 0.0225

## Data Availability

The data presented in this study are available on request from the corresponding author due to privacy.

## Appendix A

**Table A1:** Overview of label categories and examples of labeled areas.

Original Image	Label Number	Label Name	Label Area Diagram
	{0}	Background	(White)
	{1}	Green Space	
	{2}	Building	
	{3}	Road	
	{4}	Water Area	

**Table A2:** Outputs of the main evaluation metrics.

Model	Fold	Loss	Dice	mIoU	PA	mPA	Focal	Tversky
GreenViT + Green Head	F1	0.2253	0.9342	0.8773	0.9309	0.9350	0.1218	0.1323
	F2	0.1218	0.9603	0.9240	0.9590	0.9613	0.0416	0.0835
	F3	0.1133	0.9633	0.9295	0.9628	0.9643	0.0387	0.0818
	F4	0.0780	0.9648	0.9323	0.9651	0.9654	0.0361	0.0708
	F5	0.0725	0.9673	0.9370	0.9674	0.9681	0.0326	0.0635
GreenViT + FPN	F1	0.2849	0.9245	0.8606	0.9205	0.9250	0.1228	0.2110
	F2	0.2279	0.9488	0.9032	0.9460	0.9507	0.0573	0.1740
	F3	0.0922	0.9545	0.9135	0.9530	0.9563	0.0532	0.0882
	F4	0.0760	0.9594	0.9224	0.9591	0.9601	0.0445	0.0801
	F5	0.0679	0.9635	0.9299	0.9630	0.9642	0.0393	0.0697
Swin-T + FPN	F1	0.2736	0.9103	0.8371	0.9059	0.9112	0.1361	0.1947
	F2	0.2056	0.9345	0.8783	0.9317	0.9371	0.0836	0.1333
	F3	0.1901	0.9412	0.8899	0.9398	0.9433	0.0716	0.1241
	F4	0.1793	0.9458	0.8981	0.9459	0.9478	0.0625	0.1200
	F5	0.1685	0.9512	0.9077	0.9506	0.9534	0.0564	0.1026
SegFormer -B0(HF) + FPN	F1	0.3994	0.8626	0.7611	0.8561	0.8655	0.2021	0.3223
	F2	0.3440	0.8912	0.8060	0.8850	0.8906	0.1446	0.2769
	F3	0.3221	0.9011	0.8222	0.8976	0.9041	0.1238	0.2582
	F4	0.3068	0.9071	0.8321	0.9061	0.9078	0.1163	0.2477
	F5	0.2937	0.9155	0.8459	0.9132	0.9178	0.1051	0.2415

**Table A3:** GreenViT + Green Head: efficiency-normalized metrics with Green class under five-fold cross-validation.

Fold	mIoU/GFLOPs	mIoU/GB	mIoU × FPS	IoU_Green/GFLOPs	IoU_Green × FPS
F1	0.0049	0.3787	0.0877	0.0052	0.0927
F2	0.0052	0.3988	0.0924	0.0054	0.0963
F3	0.0052	0.4012	0.0930	0.0054	0.0968
F4	0.0052	0.4024	0.0932	0.0055	0.0969
F5	0.0053	0.4044	0.0937	0.0055	0.0970

**Table A4:** GreenViT + FPN: efficiency-normalized metrics with Green class under five-fold cross-validation.

Fold	mIoU/GFLOPs	mIoU/GB	mIoU × FPS	IoU_Green/GFLOPs	IoU_Green × FPS
F1	0.0036	0.3532	0.0861	0.0038	0.0916
F2	0.0037	0.3707	0.0903	0.0040	0.0953
F3	0.0038	0.3749	0.0914	0.0040	0.0960
F4	0.0038	0.3786	0.0922	0.0040	0.0964
F5	0.0039	0.3817	0.0930	0.0040	0.0966

**Table A5:** Swin-T + FPN: efficiency-normalized metrics with Green class under five-fold cross-validation.

Fold	mIoU/GFLOPs	mIoU/GB	mIoU × FPS	IoU_Green/GFLOPs	IoU_Green × FPS
F1	0.0351	3.2692	0.1674	0.0381	0.1818
F2	0.0368	3.4301	0.1757	0.0395	0.1884
F3	0.0373	3.4754	0.1780	0.0399	0.1902
F4	0.0377	3.5075	0.1796	0.0401	0.1912
F5	0.0381	3.5449	0.1815	0.0402	0.1918

**Table A6:** SegFormer-B0(HF) + FPN: efficiency-normalized metrics with Green class under five-fold cross-validation.

Fold	mIoU/GFLOPs	mIoU/GB	mIoU × FPS	IoU_Green/GFLOPs	IoU_Green × FPS
F1	0.0483	4.6144	0.1522	0.0535	0.1686
F2	0.0512	4.8866	0.1612	0.0565	0.1780
F3	0.0522	4.9848	0.1644	0.0578	0.1822
F4	0.0528	5.0448	0.1664	0.0583	0.1836
F5	0.0537	5.1285	0.1692	0.0587	0.1848

**Table A7:** Per-class results of the four models under five-fold validation.

Model	Fold	Building IoU%	Road IoU%	Water IoU%	Green IoU%
GreenViT + Green Head	F1	84.1	86.8	91.8	92.9
	F2	89.8	91.8	94.5	96.3
	F3	90.1	92.4	94.8	96.8
	F4	90.3	93.1	94.9	96.9
	F5	91.0	93.3	95.3	97.0
GreenViT + FPN	F1	82.1	85.0	90.7	91.8
	F2	87.3	89.1	93.5	95.3
	F3	88.3	90.3	93.8	96.0
	F4	89.3	91.7	94.3	96.4
	F5	90.3	92.3	94.9	96.7
Swin-T + FPN	F1	78.0	82.2	89.7	90.9
	F2	82.8	86.7	92.3	94.3
	F3	83.7	88.0	93.1	95.2
	F4	84.5	89.4	93.5	95.6
	F5	86.1	89.9	94.2	95.9
SegFormer -B0(HF) + FPN	F1	69.6	74.9	83.8	84.3
	F2	74.8	78.8	87.3	89.0
	F3	78.4	80.3	88.3	91.2
	F4	75.9	82.2	89.1	91.8
	F5	78.1	82.9	90.2	92.5
